# Elraglusib (formerly 9-ING-41) possesses potent anti-lymphoma properties which cannot be attributed to GSK3 inhibition

**DOI:** 10.1186/s12964-023-01147-8

**Published:** 2023-06-14

**Authors:** Josh T. Coats, Sudhir Tauro, Calum Sutherland

**Affiliations:** 1 Division of Cellular and Systems Medicine, School of Medicine, University of Dundee, Ninewells Hospital and Medical School, Dundee, UK; 2 Division of Molecular and Clinical Medicine, School of Medicine, University of Dundee, Ninewells Hospital and Medical School, Dundee, UK

**Keywords:** Elraglusib, 9-ING-41, GSK3, Lymphoma

## Abstract

Elraglusib (formerly 9-ING-41) is an ATP-competitive inhibitor of glycogen synthase kinase-3β (GSK3β) undergoing clinical trials for the treatment of various cancers including non-Hodgkin lymphoma (NHL). The drug reduces proliferation of several NHL cell lines and has efficacy in xenograft models of the disease. To confirm the importance of its action on GSK3β, we treated 3 lymphoma cell lines with selective, structurally distinct GSK3 inhibitors: CT99021, SB216763, LY2090314, tideglusib, and elraglusib. Stabilization of β-catenin and reduced phosphorylation of CRMP2, two validated targets of GSK3, were used as functional read-outs for GSK3 inhibition. CT99021, SB216763, and LY2090314 failed to reduce proliferation or viability in any cell line at concentrations that stabilized β-catenin and reduced CRMP2 phosphorylation. There was partial reduction of CRMP2 phosphorylation but no significant effect on β-catenin at cytotoxic doses of elraglusib. There was no indication of GSK3 inhibition at doses of tideglusib that affected cell viability and apoptosis. Cell-free kinase screening confirmed several other targets of elraglusib, distinct from the GSK3 inhibitors with no anti-lymphoma actions, including PIM kinases and MST2. These data question GSK3 as the target of elraglusib in lymphoma, and hence the utility of GSK3 expression as a ‘stand-alone’, therapeutic biomarker in NHL.

Video Abstract

**Supplementary Information:**

The online version contains supplementary material available at 10.1186/s12964-023-01147-8.

## Main text

### Introduction

Despite improvements in outcomes, high-grade B- and T-cell non-Hodgkin lymphoma (NHL) represent a significant area of unmet therapeutic need, particularly in the context of relapsed or refractory disease [1, 2].

Glycogen synthase kinase 3 (GSK3) is a ubiquitously expressed, serine/threonine kinase involved in diverse cellular functions [3–5]. Two paralogs, GSK3α and GSK3β, are expressed from independent genes [6]. GSK3 dysregulation is found in numerous cancers and higher GSK3 expression correlates with poorer survival in NHL [7, 8]. Several GSK3 inhibitors are commercially available and in vitro cytotoxicity has been observed in chronic lymphocytic leukemia (CLL), acute myeloid leukemia (AML), and chemotherapy-resistant NHL [9–11].

A recently developed GSK3 inhibitor, elraglusib (formerly 9-ING-41), is particularly noteworthy for its anti-lymphoma effects in both B- and T-cell NHL lines and immunocompromised mouse patient-derived lymphoma xenograft models [8, 12].

Elraglusib has now progressed to early-phase clinical trials for several malignant conditions, including lymphoma, with clinical efficacy reported in case studies of refractory adult T-cell leukemia/lymphoma [13, 14]. The therapeutic rationale for GSK3 inhibition in lymphoma is largely based on these early elraglusib data together with GSK3 gene knock-out models and differences in GSK3 expression between lymphoma tissue and normal lymphocytes [8, 12]. Therefore there are limited data to indicate that cytotoxicity in lymphoma can be achieved solely through the targeted pharmacological inhibition of GSK3. To clarify that the cytotoxicity observed with elraglusib is a consequence of GSK3 inhibition we investigated a panel of structurally diverse GSK3 inhibitors, including elraglusib, in both B- and T-cell NHL cell lines and assessed their effects on cell viability, apoptosis, and cellular GSK3 inhibition [4].

## Methods

### Compounds

Doxorubicin, SB216763, elraglusib (9-ING-41) and tideglusib were purchased from Cambridge Bioscience, LY2090314 from Stratech, CT99021 was synthesized as previously described [15]. All compounds were dissolved in DMSO (dimethyl sulfoxide).

### Cell culture

HS-Sultan cells were gifted from Dr Banerjee, University of Dundee. Karpas-299 cells were purchased from Public Health England and HH cells from American Type Culture Collection. Cells were grown in RPMI-1640 media containing glycine, 10% fetal bovine serum supplemented with 100 units/ml penicillin, 100 µg/ml streptomycin (all Gibco).

To measure changes in protein expression, cells in the exponential phase of growth were cultured in serum-free media overnight, prior to 4 h of drug treatment. For PARP (Poly [ADP-ribose] polymerase) cleavage assays, cells were cultured in serum-containing media for 24 h prior to protein extraction [16].

### Immunoblotting

Expression of β-catenin and phosphorylation of collapsin response-mediator protein 2 (CRMP2) were used as functional read-outs of GSK3 inhibition and PARP cleavage for apoptosis, all measured by immunoblotting. Rabbit anti-PARP (9542), rabbit anti-CRMP2 (9393) and rabbit anti-β-catenin (9562) were from Cell Signaling. Mouse anti-actin (A3853) was from Sigma-Aldrich. Sheep anti-phospho-CRMP2 (Thr509/514) was made in-house as described previously [17].

Anti-rabbit (Licor Goat anti-Rabbit (926–32211)) and anti-mouse (Thermofisher Invitrogen Goat anti-Mouse (A21057)) secondary antibodies were used at 1:10,000. Rabbit anti-sheep HRP (horseradish peroxidase) secondary (ThermoFisher 31480) was used at 1:2500. All antibodies were diluted in 1% BSA in TBS-T. Cell lysates were prepared for SDS-PAGE (sodium dodecyl sulfate–polyacrylamide gel electrophoresis) as previously described [18], and protein quantified by Bradford (BioRad). Blot analysis was on either the LICOR-Odyssey Scanner (β-catenin and β-actin) or on film using ECL (enhanced chemiluminescence) (Phospho-CRMP2), then ImageStudio Lite 5.2 and Graphpad Prism, with one-way ANOVA and *p* =  < 0.05 for statistical significance.

### Cell viability

This was assessed using CellTiter 96® Aqueous One Solution Cell Proliferation Assay Kit (Promega) according to manufacturer’s instructions. In brief, 8000 cells/well were seeded into 96-well plates. Compounds were incubated with cells for 72 h prior to the addition of MTS (3-(4,5-dimethylthiazol-2-yl)-5-(3-carboxymethoxyphenyl)-2-(4-sulfophenyl)-2H-tetrazolium) reagent for 2 h followed by absorbance assessment at 450 nM by Modulus Microplate reader. Analysis was performed using Graphpad Prism 9.

### Kinase assays

^33^P cell-free screens were performed by the MRC Protein Phosphorylation Unit, University of Dundee as previously described [19].

## Results and discussion

**Fig. 1:**
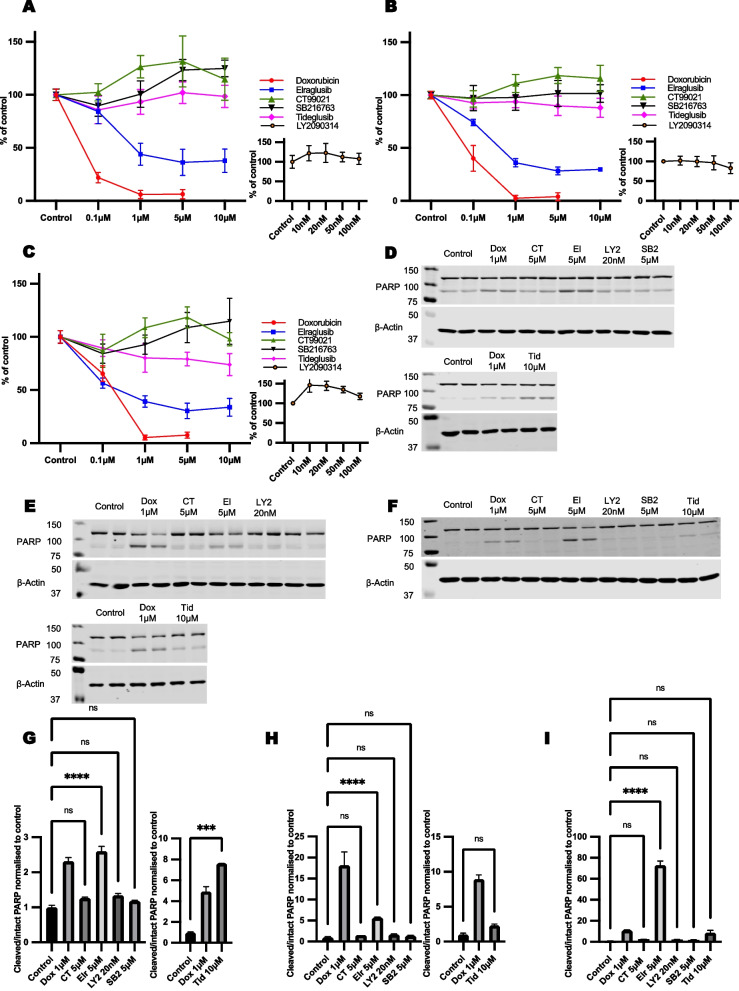
Effect of various GSK3 inhibitors on lymphoma cell viability and PARP cleavage: HS-Sultan (**A**,**D**), Karpas-299 (**B**,**E**), and HH (**C**,**F**) cells were exposed to a panel of small molecule GSK3 inhibitors. Doxorubicin, an anthracycline chemotherapeutic, was used as a positive control. Cell viability assessments were performed using the MTS assay following a 72-h exposure to the compounds. PARP cleavage assays (marker of apoptosis) were performed on lysates generated after 24 h exposure to compounds. Quantification of PARP blots from 3 separate experiments is provided for HS-Sultan (**G**), Karpas-299 (**H**), and HH (**I**) cells

Elraglusib (1-5 µM) reduced cell viability and induced apoptosis in both B- and T-cell NHL cell lines in keeping with its reported anti-lymphoma properties (Fig. 1). If the inhibition of GSK3 alone was sufficient to explain this property of elraglusib then we would expect structurally unrelated, selective inhibitors of GSK3 to have similar anti-lymphoma actions. We, therefore, investigated the effects of the GSK3 inhibitors, tideglusib, CT99021, SB216763 and LY2090314.

Tideglusib (5-10 µM) produced a weak reduction in cell viability and only induced apoptosis in one of three cell lines, while none of the other GSK3 inhibitors reduced cell viability or induced apoptosis at concentrations previously reported to result in GSK3 inhibition (Fig. 1).

**Fig. 2:**
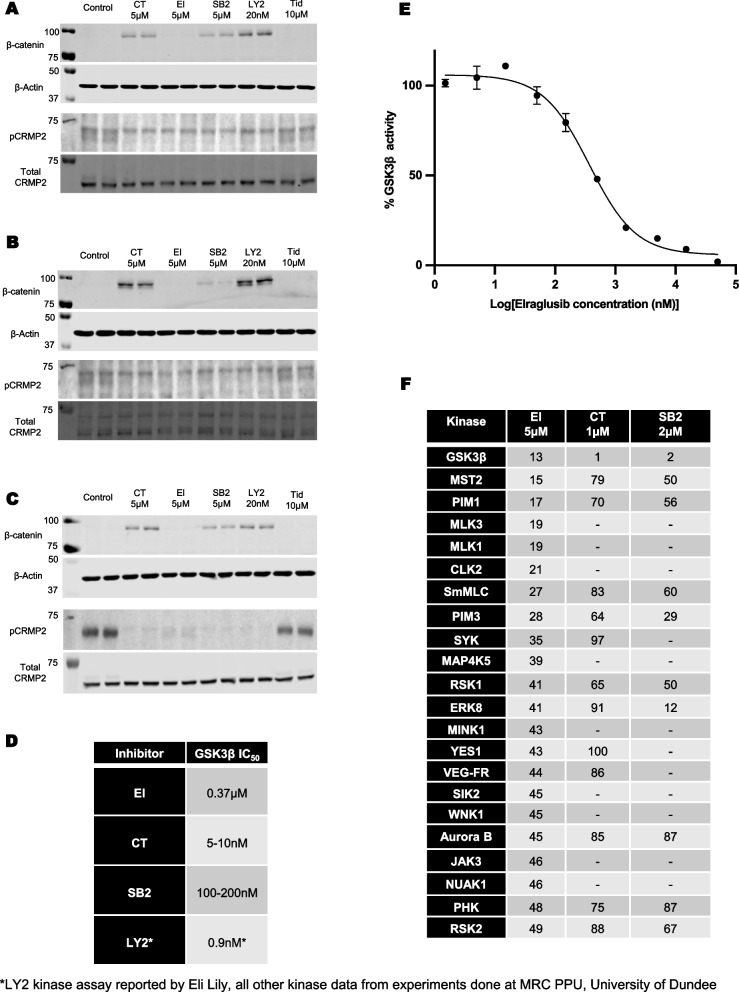
Analysis of GSK3 substrate regulation in lymphoma cells: HS-Sultan (**A**), Karpas-299 (**B**), and HH (**C**) cells were exposed to the compounds for 4 h prior to cell lysis and western blotting for the GSK3 substrates β-catenin and phospho-CRMP2, using b-actin and total CRMP2 as loading controls. **D** IC_50_ determination (0.37 µM) in an in vitro GSK3β assay. **E** Comparison of reported IC_50_ measures for the GSK3 inhibitors used in this study. **F** The 22 kinases which were inhibited more than 50% by 5 µM elraglusib in a cell-free kinase screen (100 kinases) performed within the MRC PPU at the University of Dundee. Available published data for CT99021 and SB216763 are given as comparison against these kinases in the same assay conditions. Data are ‘remaining kinase activity’ relative to DMSO carrier control and are the average of triplicate assays

CRMP2 and β-catenin are validated targets for GSK3, with β-catenin phosphorylation promoting degradation of the protein [4, 20, 21]. CT99021, SB216763 and LY2090314 all significantly stabilized β-catenin protein and reduced CRMP2 phosphorylation in every cell line at concentrations that did not alter cell viability within 4 h (Fig. 2A-C). The relative strength of these effects was consistent with reported in vitro GSK3 inhibitory potency (LY > CT > SB, Fig. 2D). Neither elraglusib nor tideglusib increased β-catenin levels in any cell line (even at 5-10 µM), although elraglusib did partly reduce the phosphorylation of CRMP2 at 5 µM in one line (Fig. 2A-C). The effects of CT99021 and LY2090314 on β-catenin were sustained for 24 h (data not shown), while the action of SB216763 declined by 24 h. Longer incubations up to 24 h with elraglusib or tideglusib did not result in any significant actions on β-catenin stability (data not shown). We confirmed the in vitro IC_50_ of our batch of elraglusib against GSK3 (0.37 µM, Fig. 2E). Therefore, the lack of correlation between the regulation of cellular GSK3 substrates and altered cell viability or apoptosis implies that a GSK3-independent action of elraglusib is responsible for its anti-lymphoma actions.

Next, we examined the in vitro sensitivity of over 100 different kinases to elraglusib and found that 22 kinases were inhibited > 50% at 5 µM (Fig. 2F). Elraglusib had potent action against GSK3b (87% inhibition at 5 µM), but equivalent potency against PIM (proto-oncogene serine/threonine-protein kinase) kinases 1 and 3, and MST2 (serine/threonine-protein kinase 3), also known as STK3. These kinases were not as sensitive to CT99021 or SB216763 (Fig. 2F) and are examples of differential off-target effects of elraglusib, worthy of further study. While this data emphasizes the need to consider other mechanism(s) of action for elraglusib it is not unusual for clinically useful drugs to have additional off-target effects, and of course, all of the known inhibitors of GSK3 have additional off-target effects.

Hence our data provide two lines of argument to suggest the anti-lymphoma action of elraglusib is not a consequence of GSK3 inhibition. Firstly, several other, more potent inhibitors of GSK3 do not alter the proliferation of lymphoma cells despite significant GSK3 inhibition. Secondly, validated targets of GSK3 action are relatively weakly inhibited by elraglusib (compared to other GSK3 inhibitors) at concentrations that generate changes in cell viability.

These are important observations as GSK3 is increasingly recognized as a potential therapeutic target in hematological malignancy. Structurally distinct GSK3 inhibitors can induce apoptosis in patient-derived CLL cells, reduce proliferation in AML cell lines and re-sensitize *TP53*-mutant Burkitt lymphoma cell lines to anthracycline chemotherapy [9–11]. Since GSK3 RNA/protein levels are higher within lymphoma cells in comparison to normal lymphocytes, and *Wu *et al. were unable to generate GSK3β knockouts in four of five lymphoma cell lines, an intrinsic dependence upon GSK3β for lymphoma survival was suggested [8]. These data led to the assumption that the efficacy of elraglusib in B- and T-cell lymphoma cell lines and patient-derived lymphoma xenograft models was due to GSK3 inhibition [8]. However, our studies demonstrate that GSK3 inhibition is unlikely to explain these actions of elraglusib. This compound inhibits additional kinases with similar potency to GSK3 including PIM kinases that are implicated in lymphomagenesis and are frequently overexpressed in human lymphomas [22–24]. Similarly, MST2 is a key member of the Hippo signaling pathway with putative roles in several cancers, including AML [25].

In conclusion, we re-affirm the anti-lymphoma effects of elraglusib but argue that this action cannot be attributed to GSK3 inhibition. Hence, we caution against the assumption that selective GSK3 inhibitors will have clinical benefit in lymphoma and against GSK3 activity as a mechanistic biomarker.

## Supplementary Information

**Additional file 1.**

## Data Availability

The datasets used and/or analyzed during the current study are available from the corresponding author on reasonable request. The raw data from the kinase screen for elraglusib will be available after publication on the MRC PPU website.

## Abbreviations

AML: Acute myeloid leukemia
CLL: Chronic lymphocytic leukemia
CRMP2: Collapsin response-mediator protein 2
DMSO: Dimethyl sulfoxide
ECL: Enhanced chemiluminescence
GSK3: Glycogen synthase kinase 3
HRP: Horseradish peroxidase
MST2/STK3: Serine/threonine-protein kinase 3
MTS: 3-(4,5-Dimethylthiazol-2-yl)-5-(3-carboxymethoxyphenyl)-2-(4-sulfophenyl)-2H-tetrazolium
NHL: Non-Hodgkin lymphoma
PARP: Poly (ADP-ribose) polymerase
PIM: Proto-oncogene serine/threonine-protein kinase
SDS-PAGE: Sodium dodecyl sulfate–polyacrylamide gel electrophoresis
