# Supplementary material for: Elraglusib (formerly 9-ING-41) possesses potent anti-lymphoma properties which cannot be attributed to GSK3 inhibition
Source: Cell Commun Signal. 2023 Jun 14;21:131. doi: 10.1186/s12964-023-01147-8 (PMC10265916; doi:10.1186/s12964-023-01147-8)

Original Blots used to make Figure 1

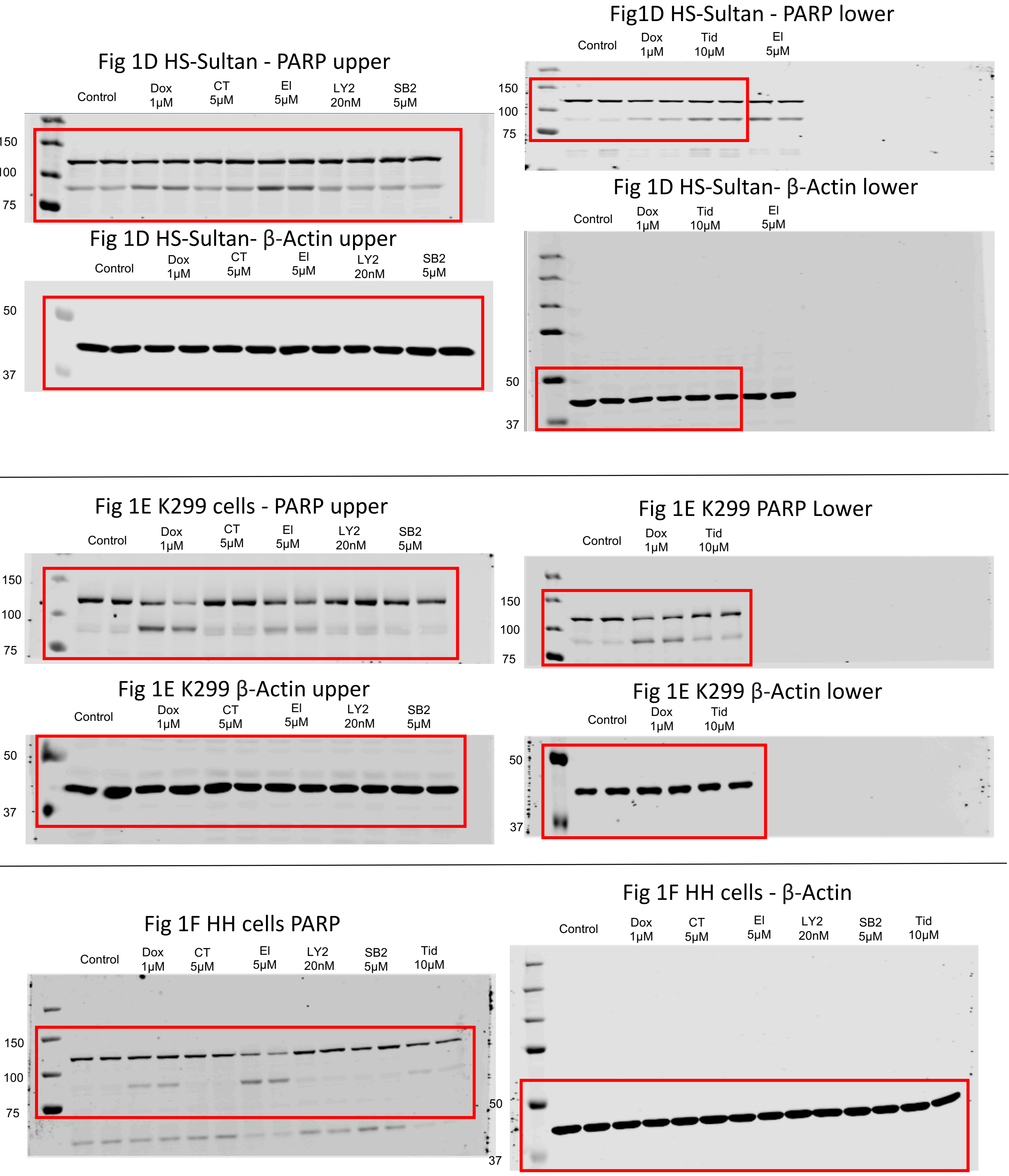

Figure 1. Effect of various GSK3 inhibitors on lymphoma cell viability and PARP cleavage: HS-Sultan (D), Karpas-299 (E), and HH (F) cells were exposed to a panel of small molecule GSK3 inhibitors (described in text) or Doxorubicin. PARP cleavage assays (marker of apoptosis) were performed on lysates generated after 24 hours exposure to compounds, with b-actin as a loading control.

Original blots used to make Figure 2 (next 2 slides)

Fig 2A HS-Sultan - β-Catenin

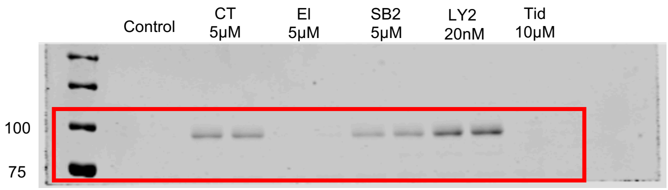

Fig 2A HS-Sultan - pCRMP2

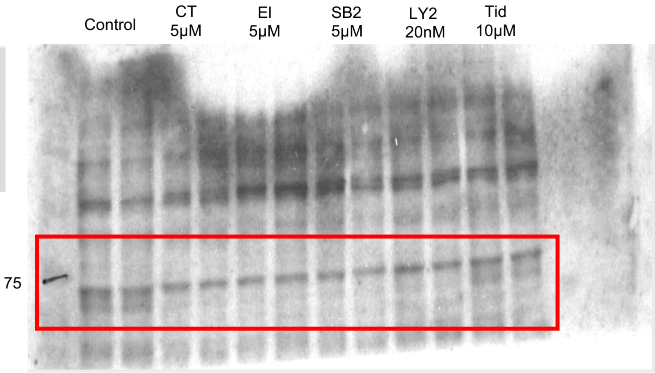

Fig 2A HS-Sultan – total CRMP2

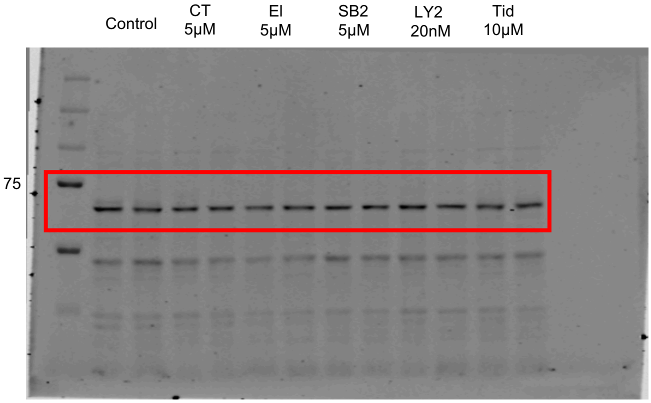

Fig 2A HS-Sultan- β-Actin

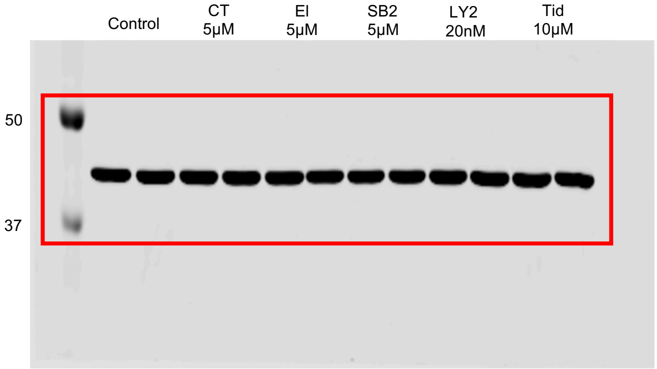

Figure 2: Analysis of GSK3 substrate regulation in lymphoma cells: HS-Sultan (A), Karpas-299 (B), and HH (C) cells were exposed to the compounds as indicated for 4 hours prior to cell lysis and western blotting for the GSK3 substrates β-catenin and phospho-CRMP2, using β-actin or total CRMP2 as loading controls.

Original data used in Figure 2 (2 of 2)

Fig 2B K299 - β-Catenin

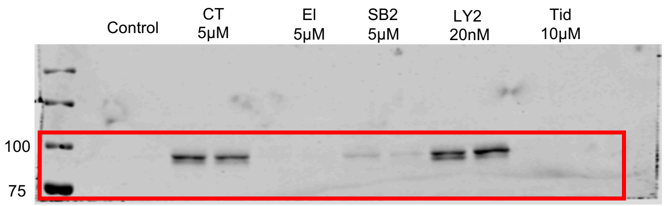

Fig 2B K299 total CRMP2

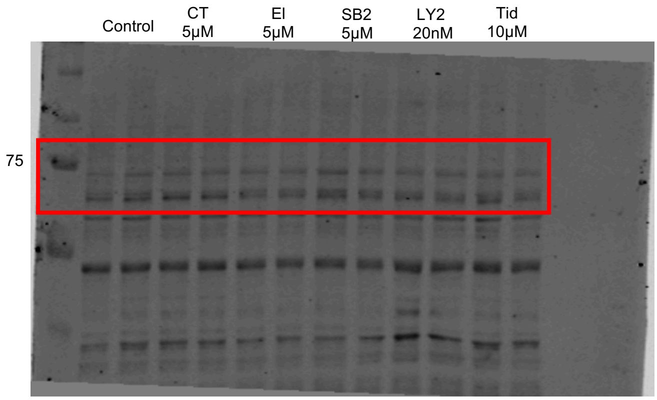

Fig 2B K299 - pCRMP2

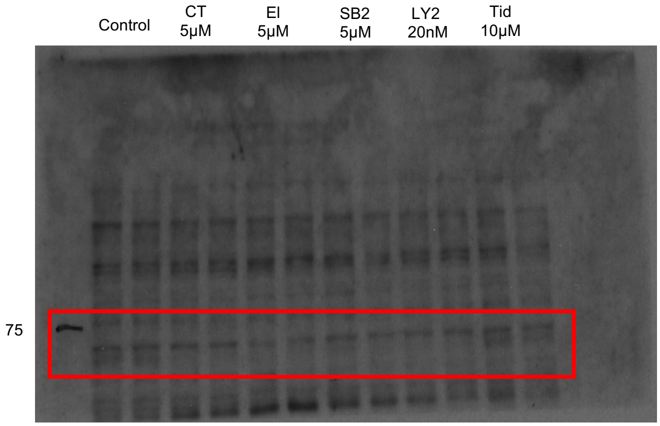

Fig 2B K299 - β-Actin

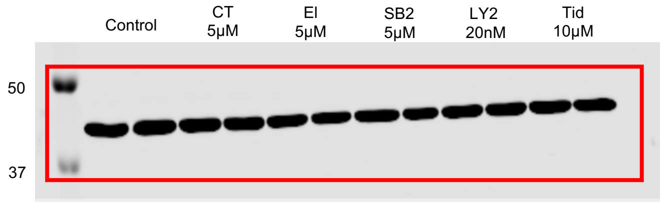

Fig 2C HH - β-Catenin

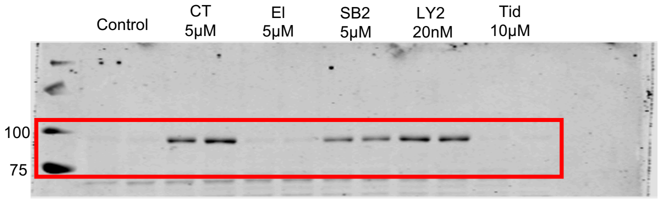

Fig 2C HH total CRMP2

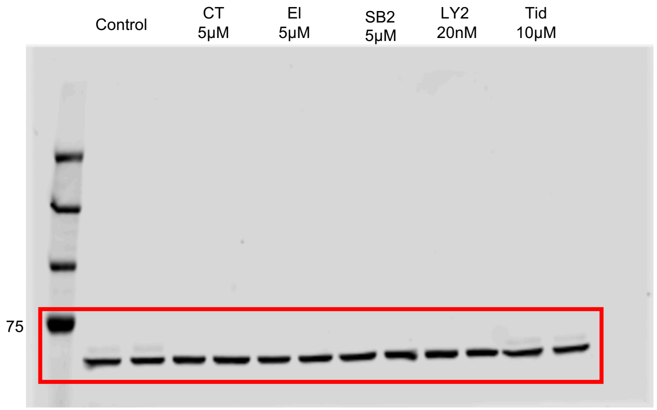

Fig 2C HH - pCRMP2

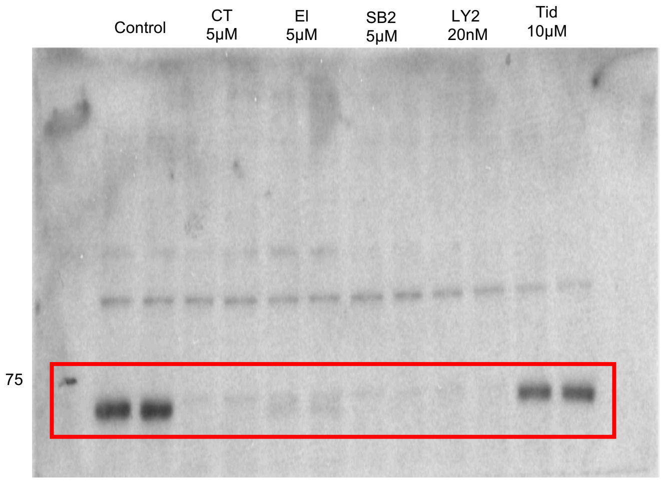

Fig 2C HH - β-Actin

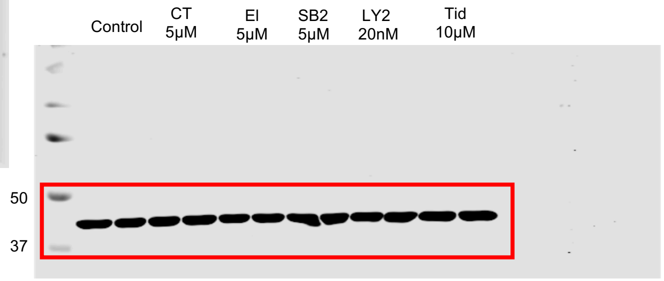

Supplement: Supplementary file 2 — Additional file 1. [file 12964_2023_1147_MOESM1_ESM.pdf]
